# Pharmacokinetics and coagulation biomarkers in children and adults with hemophilia A receiving emicizumab prophylaxis every 1, 2, or 4 weeks

**DOI:** 10.1016/j.rpth.2023.102306

**Published:** 2023-12-29

**Authors:** Anna Kiialainen, Joanne I. Adamkewicz, Claire Petry, Johannes Oldenburg, Steven W. Pipe, Guy Young, Johnny Mahlangu, Michaela Lehle, Markus Niggli, Giancarlo Castaman, Víctor Jiménez-Yuste, Midori Shima, Claude Négrier, Christophe Schmitt

**Affiliations:** 1 F. Hoffmann-La Roche Ltd, Basel, Switzerland; 2 Genentech, Inc, South San Francisco, California, USA; 3 University of Bonn, Bonn, Germany; 4 University of Michigan, Ann Arbor, Michigan, USA; 5 Children’s Hospital Los Angeles, Keck School of Medicine, University of Southern California, Los Angeles, California, USA; 6 University of the Witwatersrand and National Health Laboratory Service, Johannesburg, South Africa; 7 Careggi University Hospital, Florence, Italy; 8 Department of Hematology, La Paz University Hospital-IdiPAZ, Autónoma University, Madrid, Spain; 9 Nara Medical University, Kashihara, Japan; 10 Louis Pradel University Hospital, Lyon, France

**Keywords:** biomarkers, emicizumab, hemophilia A, pharmacodynamics, pharmacokinetics

## Abstract

**Background:**

Emicizumab is a bispecific antibody that bridges activated factor (F)IX and FX, mimicking the function of missing activated FVIII and thus improving hemostasis in people with hemophilia A. The efficacy and safety of emicizumab were demonstrated in 4 phase III clinical trials (HAVEN 1-4).

**Objectives:**

Here, we describe pharmacokinetics (PKs), pharmacodynamics (PDs), and exploratory safety biomarkers in HAVEN 1 to 4.

**Methods:**

Participants received emicizumab at a loading dose of 3 mg/kg weekly for 4 weeks, followed by maintenance doses of 1.5 mg/kg weekly, 3 mg/kg every 2 weeks, or 6 mg/kg every 4 weeks. PKs, PDs, and safety biomarkers were assessed in samples collected at regular intervals during the trials.

**Results:**

Emicizumab plasma trough concentrations increased during the loading dose period, reaching a mean of 52.9 μg/mL (SD, 13.6 μg/mL) at week 5, and were sustained at 42.1 to 52.3 μg/mL thereafter with maintenance dosing. Activated partial thromboplastin time shortened following the first emicizumab dose. Mean FVIII-like activity and thrombin generation peak height increased to 25.2 IU/dL (SD, 6.9 IU/dL) and 115.2 nM (SD, 42.5 nM) at week 5, with levels sustained at 17 to 23 IU/dL and >116 nM thereafter, respectively. Emicizumab did not notably affect FIX or FX plasma antigen levels, prothrombin time, or concentrations of exploratory safety markers of coagulation activation (D-dimer, prothrombin fragment 1 + 2, and fibrinogen).

**Conclusion:**

In HAVEN 1 to 4, emicizumab demonstrated sustained PKs and PDs and improved coagulation parameters without affecting safety biomarkers.

## Essentials

• The efficacy and safety of emicizumab were previously demonstrated in the HAVEN 1 to 4 trials.
• Pharmacokinetics, pharmacodynamics, and safety biomarkers were evaluated in HAVEN 1 to 4.
• Emicizumab demonstrated sustained pharmacokinetics and pharmacodynamics throughout the trials.
• Factor IX/factor X plasma antigen levels, prothrombin time, and safety markers were not notably affected.

## 1 Introduction

Hemophilia A (HA) is a congenital, X-linked, recessive disorder resulting from the absence or dysfunction of coagulation factor (F)VIII, leading to lifelong bleeding tendency. Historically, FVIII replacement, given prophylactically with additional on-demand dosing during a bleeding episode, was the standard of care for HA. However, due to the short half-life of FVIII, multiple intravenous infusions per week are required to achieve the recommended trough levels for effective prophylaxis. Achieving higher trough factor levels (3%-5%) to maintain steady-state hemostasis is an important target for prophylaxis and a current challenge of standard and extended half-life clotting factor products [1].

Emicizumab is a recombinant, humanized, bispecific monoclonal antibody that bridges activated FIX (FIXa) and FX, mimicking the function of absent or deficient activated FVIII, thereby improving hemostasis in people with HA [2,3]. Emicizumab has a long half-life of approximately 30 days [4,5] and has high subcutaneous bioavailability [6], enabling subcutaneous dosing and negating the requirement for venous access. Emicizumab shares no sequence homology with FVIII [7] and can therefore restore hemostasis in people with HA with or without FVIII inhibitors [3].

During its development, the efficacy and safety of emicizumab were originally demonstrated in 4 phase III clinical trials (HAVEN 1-4: NCT02622321, NCT02795767, NCT02847637, and NCT03020160) across a broad population of people with HA, of different ages, with or without FVIII inhibitors [[8], [9], [10], [11]]. The safety and efficacy of emicizumab continue to be monitored and assessed in several ongoing phase III clinical trials, including HAVEN 5 (NCT03315455) [12], HAVEN 6 (NCT04158648) [13], and HAVEN 7 (NCT04431726) [14]. During the primary analyses of HAVEN 1 to 4, emicizumab prophylaxis resulted in marked reductions in annualized bleeding rates of treated bleeds, and the majority of participants (56%-90%) receiving emicizumab in each study did not report any treated bleeds [[8], [9], [10], [11]]. Analyses of long-term safety and efficacy data for emicizumab were consistent with the findings of the primary analyses, and indicated that people with HA treated with emicizumab continue to experience improved bleeding prevention and target joint resolution [15].

In a previous analysis of emicizumab pharmacokinetics (PKs) and pharmacodynamics (PDs) in adults with HA with FVIII inhibitors in HAVEN 1, a maintenance dose of 1.5 mg/kg weekly provided sustained therapeutic trough plasma emicizumab concentrations and PD effects throughout >16 months of follow-up (median duration of exposure to emicizumab, 60.5 weeks; range, 3.3-94.2 weeks) [16]. Based on achieved FVIII-like activity and thrombin generation (TG), emicizumab appeared to transform a severe bleeding phenotype to a mild phenotype [16]. Here, we describe PK, PD, and exploratory safety biomarker data in a larger and more diverse population of people with HA (pediatric and adult; with and without FVIII inhibitors) receiving emicizumab weekly, every 2 weeks, or every 4 weeks in the HAVEN 1 to 4 trials, with a median of 2.3 years (IQR, 1.7-4.6 years) of follow-up.

## 2 Methods

### 2.1 Study populations

The HAVEN 1 to 4 clinical trials were phase III multicenter, open-label studies. A summary of the individual study designs is shown in the Table, with inclusion and exclusion criteria, methods, and types of data collected having been reported previously [[8], [9], [10], [11]]. All adult participants provided written informed consent prior to study entry. For participants aged <18 years, informed consent was provided by a parent or legally authorized representative, along with informed assent in those aged 8 to 17 years. The relevant independent ethics committee/institutional review board at each participating institution approved each study protocol, and the studies were conducted in accordance with the principles of the Declaration of Helsinki and Good Clinical Practice [17].

**Table:** Summary of the HAVEN 1 to 4 clinical trials.

Clinical trial	Age range	Inhibitor status	Emicizumab dosing regimen	Cutoff date^a^	Number of participants^b^	Plasma sample collection schedule
HAVEN 1 [8]	≥12 y	With FVIII inhibitors	QW	December 1, 2020	113	• Before first emicizumab dose • At trough - QW for month 1 - Q2W for month 2 - Q4W for months 3-6 - Q8W from months 7 to 12 - Q12W thereafter
HAVEN 2 [9]	<12 y^c^	With FVIII inhibitors	QW, Q2W, or Q4W	November 11, 2020	88	• Before first emicizumab dose • At trough - Q2W for month 1 - Q4W for month 2-10 - Q8W from months 11-14 - Q12W thereafter
HAVEN 3 [10]	≥12 y	Without FVIII inhibitors	QW or Q2W	May 12, 2022	152	• Before first emicizumab dose • At trough - QW for month 1 - Q2W for month 2 - Q4W for months 3-6 - Q8W from months 7-12 - Q12W thereafter
HAVEN 4 [11]	≥12 y	With or without FVIII inhibitors	Q4W	June 29, 2022	41	• Before first emicizumab dose • At trough - QW for month 1 - Q4W for months 2-6 - Q12W thereafter

FVIII, factor VIII; QW, once weekly; Q2W, every 2 weeks; Q4W, every 4 weeks; Q8W, every 8 weeks; Q12W, every 12 weeks.

a Cutoff date is set to the date of the last patient’s last visit.

b Number refers to participants who contributed to the present analyses. One participant from HAVEN 1 and 1 participant from HAVEN 3 were not treated and only contributed to baseline data.

c Participants aged >12 years were eligible for inclusion if their weight was <40 kg. Three participants aged >12 years were enrolled in HAVEN 2.

Samples were collected until the last patient’s last visit for each study, resulting in the following clinical cutoff dates: December 1, 2020, for HAVEN 1; November 11, 2020, for HAVEN 2; May 12, 2022, for HAVEN 3; and June 29, 2022, for HAVEN 4.

### 2.2 Sample collection

Of the total 401 enrolled participants, 394 contributed plasma samples for PK and biomarker analyses in the HAVEN 1 to 4 trials at scheduled time points. Data from the run-in phase of the HAVEN 4 study are not included in this analysis [11]. The schedule of plasma sample collection for each study is shown in the Table.

### 2.3 PKs

Emicizumab plasma concentrations were measured using a validated enzyme-linked immunosorbent assay (ELISA) [18], performed by QPS Netherlands B.V. The lower limit of quantification was 100 ng/mL in human plasma. Assay precision and accuracy were 3.1% to 13.7% and 88.9% to 107.5%, respectively.

### 2.4 Biomarker assays

Biomarker analyses were performed by Medpace Reference Laboratories. Activated partial thromboplastin time (aPTT) was measured using the STA-PTT A kit (Diagnostica Stago). Prothrombin time (PT) was measured with the Neoplastine CI Plus kit (Diagnostica Stago). FIX and FX plasma antigen levels were measured using human FIX and FX ELISAs (Assaypro). D-dimer concentrations were measured using the STA-Liatest D-DI kit (Diagnostica Stago), and prothrombin fragment 1+2 using Enzygnost F1+2 ELISA (Siemens).

FVIII activity was measured using a chromogenic assay containing human coagulation factors (BIOPHEN FVIII; Hyphen Biomed) on Stago Evolution coagulation analyzer (Diagnostica Stago) according to the manufacturer’s instructions. Two different calibration curves constructed with BIOPHEN Plasma Calibrator (Hyphen Biomed) were used: high for samples with ≥10-IU/dL FVIII activity and low for samples with <10-IU/dL FVIII activity. FVIII activity reported for people with HA treated with emicizumab using this assay cannot be compared with, or interpreted as equivalent to, FVIII activity reported in participants treated with FVIII due to different biochemical properties of the 2 proteins [19]. Therefore, FVIII activity is referred to as “FVIII-like activity” throughout this article. It should also be noted that this assay kit used to determine FVIII activity was labeled “For Research Use Only,” and despite extensive laboratory validation, it was subject to lot-to-lot variation over the multiyear time course of these clinical studies. TG was measured with the Calibrated Automated Thrombogram (Diagnostica Stago) according to the manufacturer’s instructions and using human activated FXI as the initiator, as described by Waters et al. [20]. FluCa kit, MP reagent, thrombin calibrator, and human FXIa were all obtained from Stago. The Stago Calibrated Automated Thrombogram system used consisted of a Fluoroskan Ascent reader and Thrombinoscope software, version 5.0.0.742 (Stago). TG peak height was derived from the thrombogram.

### 2.5 FVIII inhibitors

FVIII inhibitors were analyzed using a chromogenic Bethesda assay (CBA) [21]. A chromogenic FVIII activity assay containing bovine coagulation FIXa and FX (Siemens) was used to measure residual FVIII activity in this assay [21,22]. Due to sample volume limitation, the upper limit for the CBA was censored to 45 chromogenic Bethesda unit (CBU)/mL. Values reported as >45 CBU/mL were set to 45 CBU/mL for the analysis. The cutoff value for inhibitor positivity in the CBA was 0.6 CBU/mL.

### 2.6 Data analysis

Data from all participants in the HAVEN 1 to 4 studies receiving emicizumab at a maintenance dose of 1.5 mg/kg weekly, 3 mg/kg every 2 weeks, or 6 mg/kg every 4 weeks are reported here. PK and biomarker data from the HAVEN 1 to 4 trials were pooled and split by dosing regimen, inhibitor status, or age group for specific analyses. Data from the HAVEN 1 trial reported previously (clinical cutoff date of September 8, 2017) [16] are included in this pooled analysis.

PK and biomarker data were subject to descriptive analysis. Data from participants who were uptitrated to emicizumab 3 mg/kg weekly due to suboptimal bleed control [23] (*n* = 21) were included in the descriptive statistics until uptitration but were included in the PK/PD relationship plots both before and after uptitration. Scheduled time relative to first emicizumab dose in participants who switched to emicizumab prophylaxis after completing 24 weeks on study in control arms (arm B in HAVEN 1 and arm C in HAVEN 3) was used for graphical displays.

aPTT and TG data from participants who had their blood samples drawn via central venous lines, and for whom sample contamination with heparin was suspected, were excluded.

## 3 Results

### 3.1 Population

Baseline characteristics for HAVEN 1 to 4 participants have been reported previously [[8], [9], [10], [11]]. The overall population in this analysis comprised 113 participants from HAVEN 1, 88 participants from HAVEN 2, 152 participants from HAVEN 3, and 41 participants from the expansion phase of HAVEN 4. Of these 394 participants, 89.3% were of non-Hispanic/Latino ethnicity, 9.6% were of Hispanic/Latino ethnicity, and 1.0% were of unknown ethnicity; 66.5% were of White race, 18.8% were Asian, 8.1% were Black/African American, 0.5% were Native Hawaiian/Other Pacific Islander, 0.3% were American Indian/Alaska Native, 0.5% were of multiple races, and 5.3% were of unknown race. From the total 394 participants, 2 did not receive any treatment with emicizumab and therefore contributed only to baseline samples. Excluding the 2 who did not receive treatment, all participants received a loading dose of subcutaneous emicizumab 3 mg/kg weekly for 4 weeks. Thereafter, 279, 62, and 51 participants received subcutaneous maintenance doses of 1.5 mg/kg weekly, 3 mg/kg every 2 weeks, and 6 mg/kg every 4 weeks, respectively. A documented history of high FVIII inhibitor titer (>5 BU/mL) was present for 206 participants (52.3%) at study entry; 201 had FVIII inhibitor measurements at baseline. The median exposure time excluding uptitration was 119 weeks (IQR, 87-238 weeks), with 38.3% of participants having a follow-up of >168 weeks. The median exposure time including the uptitration period was 130 weeks (IQR, 93-247 weeks), with 40.1% of participants having a follow-up period of >168 weeks.

### 3.2 PK

**Figure 1:**
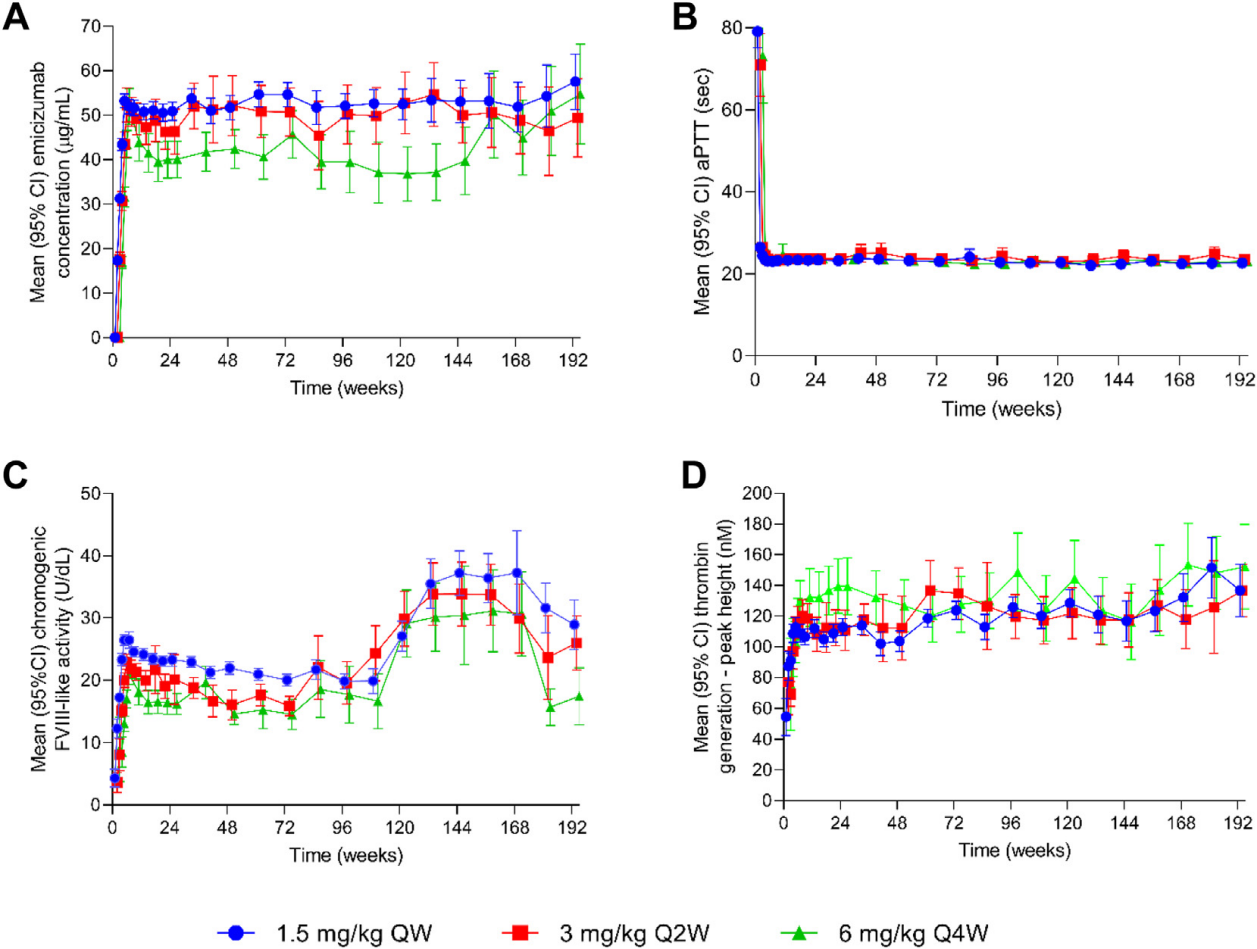
Analysis of pharmacodynamics in people with hemophilia A receiving emicizumab: (A) emicizumab trough plasma concentrations, (B) activated partial thromboplastin time (aPTT), (C) chromogenic factor (F)VIII-like activity, and (D) peak height of thrombin generation. FVIII activity was measured using a chromogenic assay containing human coagulation factors. QW, once weekly; Q2W, every 2 weeks; Q4W, every 4 weeks.

The PK analysis population consisted of 392 participants who had ≥1 postdose emicizumab concentration sample. Mean emicizumab trough concentrations during the 4 loading doses of 3 mg/kg weekly reached 52.9 μg/mL at week 5 and were sustained at mean values of 52.3 μg/mL (SD, 19.1 μg/mL), 48.4 μg/mL (SD, 20.0 μg/mL), and 42.1 μg/mL (SD, 16.5 μg/mL) thereafter, with the maintenance doses of 1.5 mg/kg weekly, 3 mg/kg every 2 weeks, and 6 mg/kg every 4 weeks, respectively (Figure 1A). Variability of emicizumab trough concentrations across the entire population, as expressed by coefficient of variation, was 30% to 40% depending on dosing regimen.

### 3.3 PD

Mean aPTT was prolonged at baseline (week 1) but shortened to below the upper limit of the reference range (23.9-40.0 seconds) by week 2 after the first dose of emicizumab. Thereafter, levels were maintained at, or just below, the reference range (Figure 1B). Thus, aPTT was shortened to within the reference range during the loading period when emicizumab concentrations had not yet reached therapeutic levels, and this shortening should not be considered as normalization of hemostasis. There was no difference in aPTT between the dosing regimens.

Mean FVIII activity at baseline, prior to emicizumab exposure, measured with the chromogenic assay containing human coagulation factors, was 5 IU/dL in participants from HAVEN 3 and 4 without FVIII inhibitors. Overall, 49% of participants had a baseline FVIII activity of <1 IU/dL, which is expected in people with severe HA. The observed FVIII activity at baseline in the other 51% of participants, all in HAVEN 3 and 4, reflects the use of FVIII products prior to study entry, as FVIII use was permitted until the second emicizumab loading dose in individuals switching from previous FVIII prophylaxis. Mean FVIII-like activity increased during the emicizumab loading doses to 25.2 IU/dL (SD, 6.9 IU/dL) at week 5 and was sustained until week 109 at approximately 23 IU/dL, 19 IU/dL, and 17 IU/dL with the maintenance doses of 1.5 mg/kg weekly, 3 mg/kg every 2 weeks, and 6 mg/kg every 4 weeks, respectively (Figure 1C). The apparent increase in FVIII-like activity after week 120 and the apparent decrease after week 168 are considered an artifact, as the increase and decrease in measured values coincided with changes in the chromogenic FVIII activity assay kit lots; there was no concomitant change in emicizumab concentration or TG. FVIII-containing plasma samples were generally used for lot-to-lot bridging studies during these analyses. After the observation of the increase in HAVEN participant sample measurements (after week 120), emicizumab-containing samples were included in the subsequent lot-to-lot bridging studies. During the bridging study coinciding with the decrease in emicizumab measurements (around week 168), the FVIII-containing samples yielded an acceptable +3% bias between kit lots while the emicizumab-containing samples yielded a −39% bias, indicating that the apparent shifts in FVIII-like activity seen in the HAVEN participant samples around weeks 120 and 168 were an assay lot change artifact specific to the emicizumab mechanism of action.

The mean TG peak height of 61 nM at baseline also reflects the use of FVIII products prior to study entry in some participants who enrolled into HAVEN 3 and 4. Forty-nine percent of participants had no detectable TG at baseline. Mean TG peak heights increased during loading doses to 115.2 nM (SD, 42.5 nM) at week 5 and were sustained above 116 nM thereafter, regardless of dosing regimen (Figure 1D). No TG data were collected in HAVEN 2 due to sample volume limitations in children.

### 3.4 PK/PD correlations

**Figure 2:**
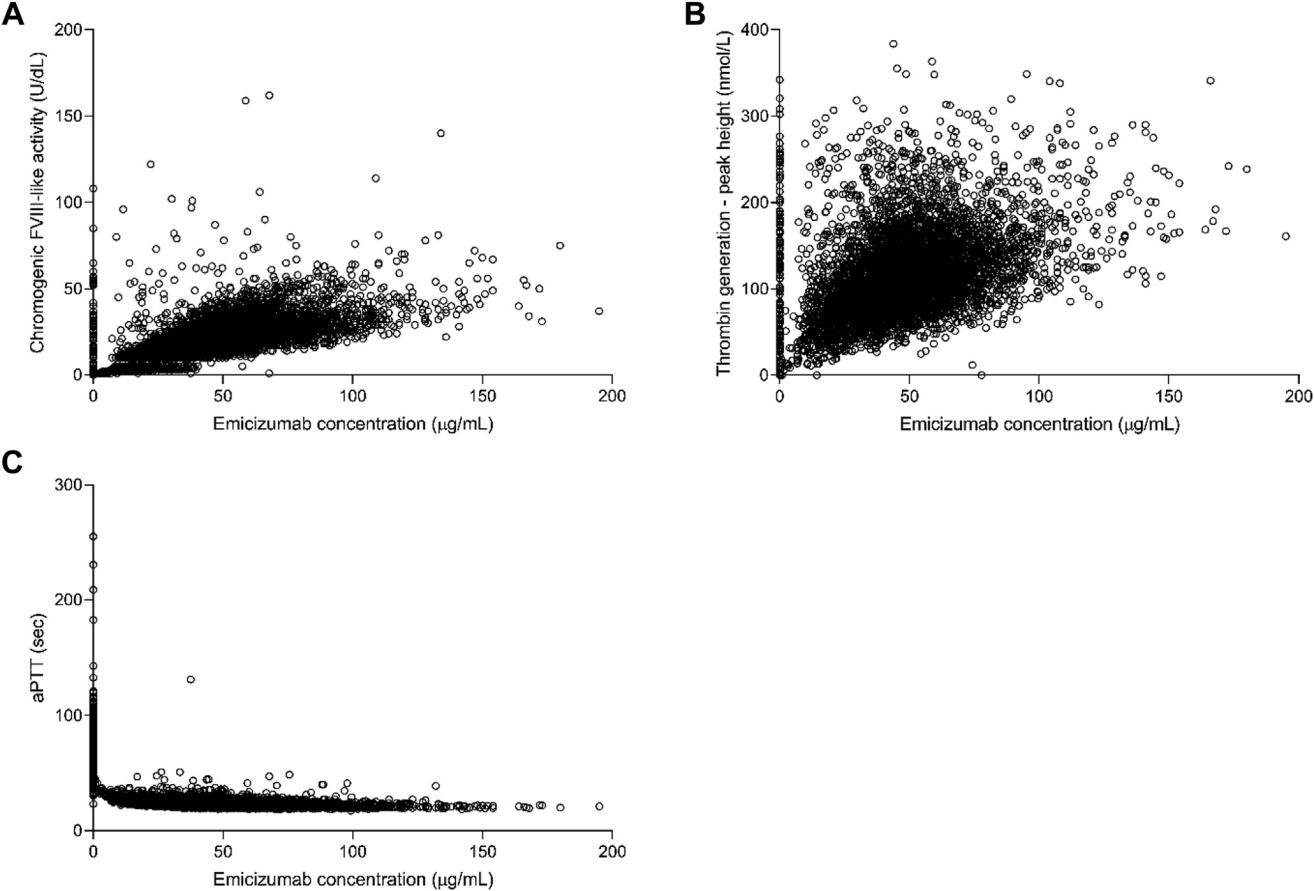
Correlation between emicizumab plasma concentrations and (A) chromogenic factor (F)VIII-like activity, (B) peak height of thrombin generation, and (C) activated partial thromboplastin time (aPTT). Gap at 10 IU/dL reflects the use of 2 different calibration curves.

**Figure 3:**
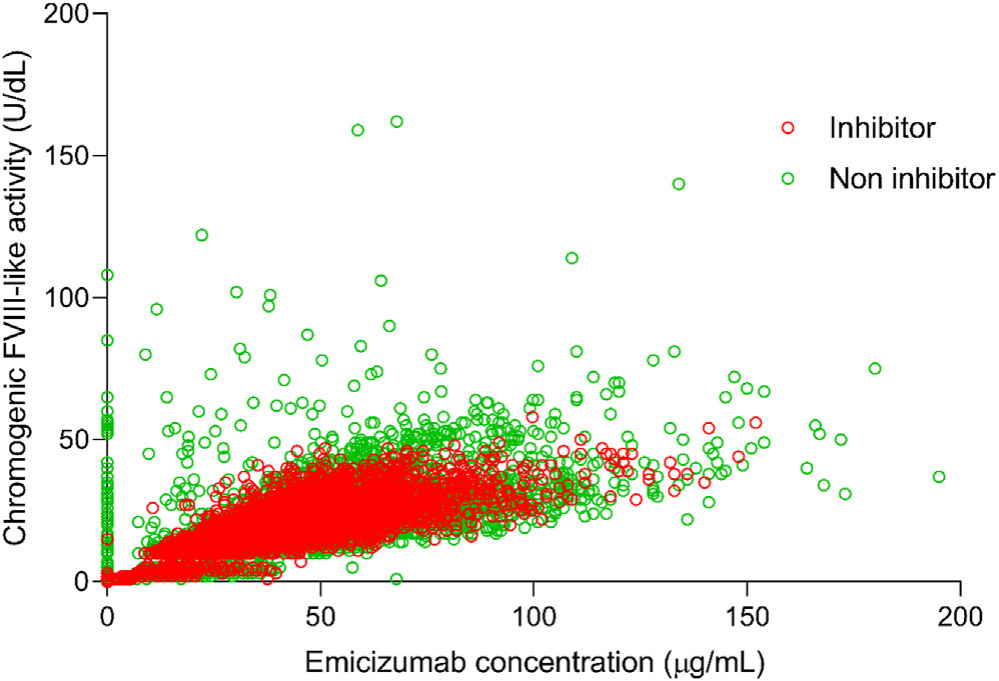
Correlation between emicizumab plasma concentrations and chromogenic factor (F)VIII-like activity between people with and without FVIII inhibitors.

Correlations between emicizumab plasma concentrations and PD biomarkers were examined. Emicizumab plasma concentrations positively correlated with chromogenic FVIII-like activity (Figure 2A) and peak height of TG (Figure 2B). Noticeably higher FVIII-like activity and TG peak height values at very low or zero emicizumab concentrations are attributed to the add-on effect of FVIII products used prior to study start and/or during the first week of emicizumab prophylaxis in some participants in HAVEN 3 and 4. Similarly, sporadic higher FVIII-like activity and TG peak height values seen in the presence of higher emicizumab concentrations are attributed to the add-on effect of FVIII products and/or recombinant activated FVII or activated prothrombin complex concentrate (aPCC) used sporadically during the studies (ie, to treat a bleed). The data gap observed at 10 IU/dL in FVIII-like activity results from the use of 2 different calibration curves, as explained above. aPTT was shortened already at very low emicizumab concentrations (>5 μg/mL), with the maximum effect achieved at concentrations >30 μg/mL (Figure 2C). Overall, similar PK/PD relationships were observed in people with HA with and without FVIII inhibitors (Figure 3).

### 3.5 Exploratory safety biomarkers

**Figure 4:**
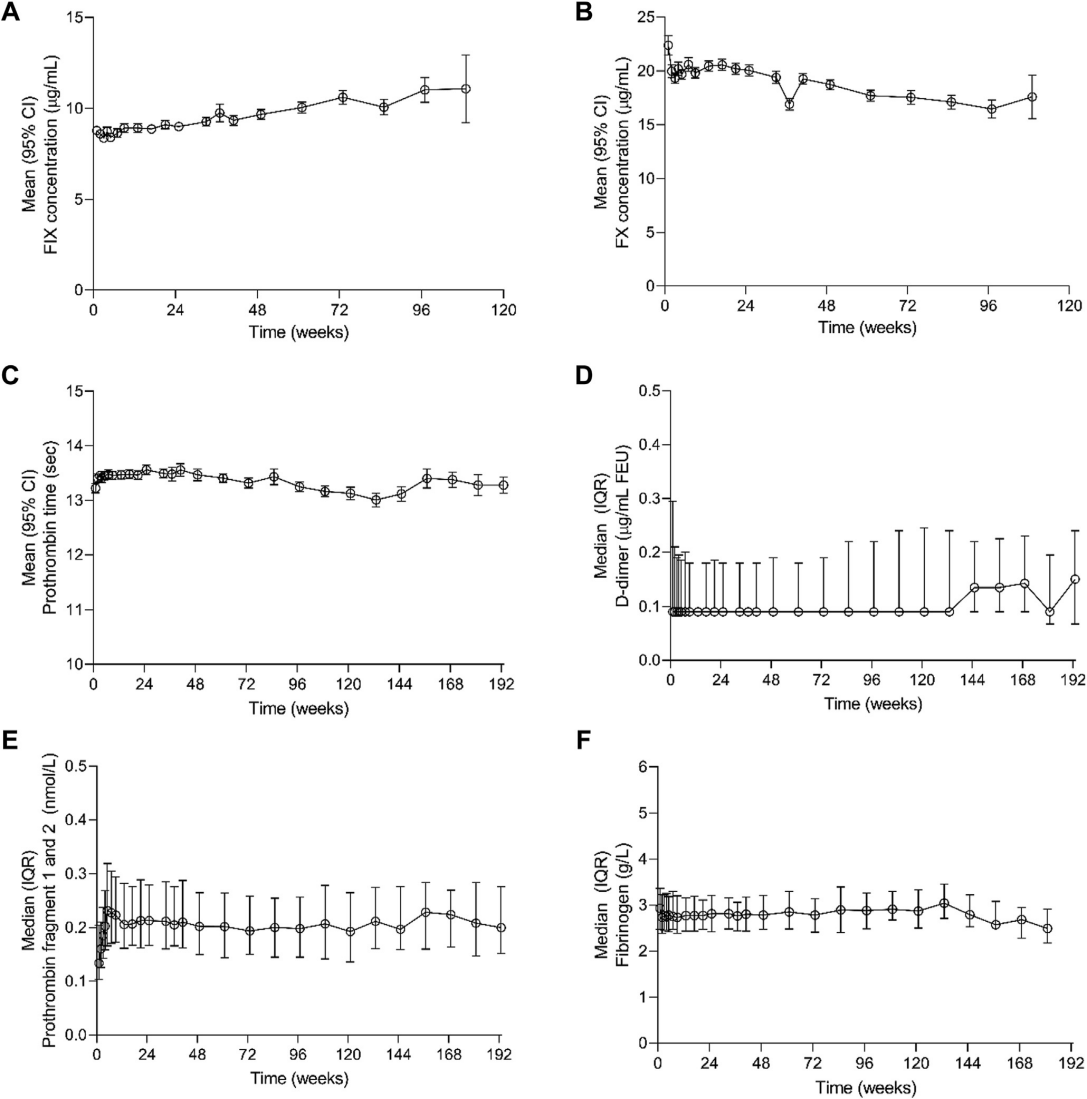
Concentrations of exploratory safety biomarkers: (A) factor (F)IX, (B) FX, (C) prothrombin time, (D) D-dimer, (E) fibrinogen, and (F) prothrombin fragment 1+2. FIX and FX plots are only presented up to 120 weeks as measurements were discontinued after that point due to no changes being observed in any of the studies up to 109 weeks of follow-up. CI, confidence interval; FEU, fibrinogen equivalent unit; FIX, factor IX; FX, factor X; IQR, interquartile range; PT, prothrombin time.

Emicizumab treatment did not have a significant impact on concentrations of its binding targets FIX or FX (Figure 4A, B), which remained stable throughout the study period. FIX and FX measurements were discontinued in August 2018 as no changes were observed in any studies up to 109 weeks of follow-up. Similarly, emicizumab treatment did not have a significant impact on PT (Figure 4C) or markers of activated coagulation: concentrations of D-dimer (Figure 4D) and fibrinogen (Figure 4E) remained stable throughout emicizumab treatment. A small, nonsignificant increase that remained within the normal range was seen in prothrombin fragment 1+2 during the emicizumab loading dose period (Figure 4F). It then remained stable from week 5 throughout emicizumab treatment.

### 3.6 FVIII inhibitors

**Figure 5:**
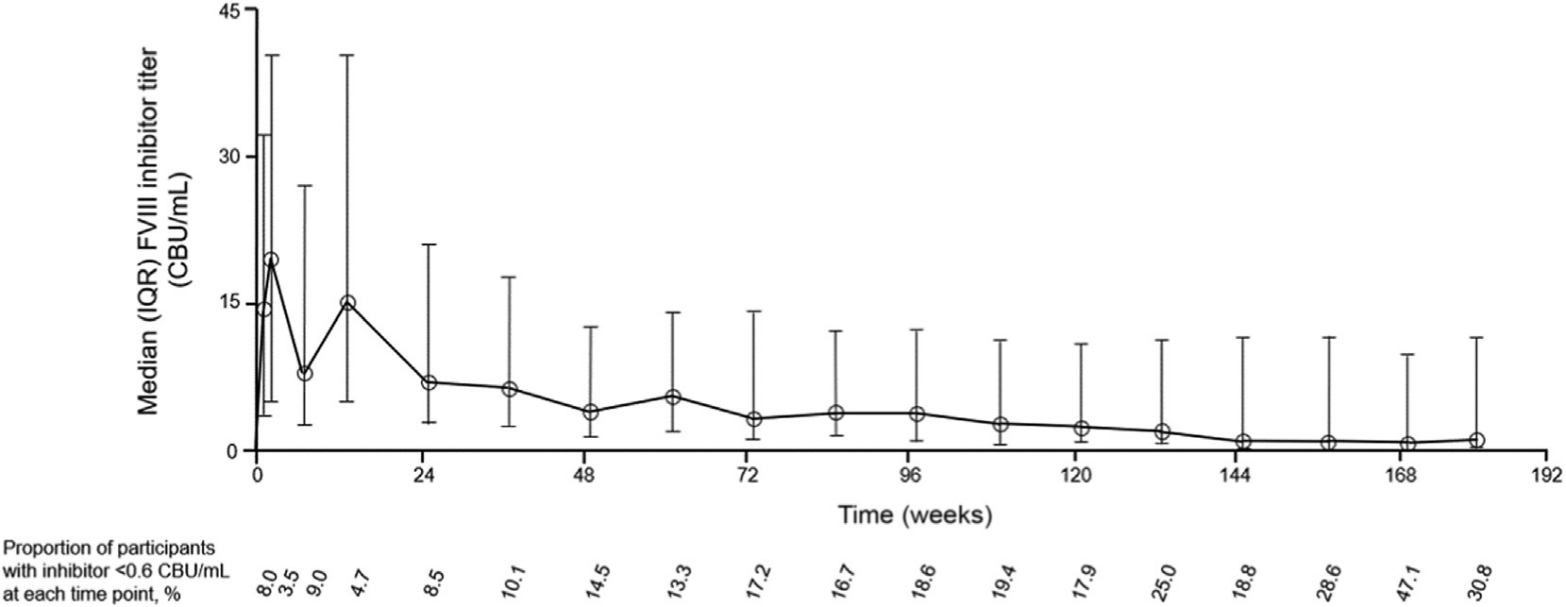
Factor (F)VIII inhibitor titer over time in participants with inhibitors. CBU, chromogenic Bethesda unit; FVIII, factor VIII; IQR, interquartile range.

Participants enrolled in HAVEN 1 and 2 were required to have a documented history of high-titer (>5 BU/mL) inhibitors but were not required to have a high titer at the time of enrollment. HAVEN 4 enrolled people with HA with and without FVIII inhibitors. The mean FVIII inhibitor titer was 19.0 CBU/mL at baseline (*n* = 201) and declined over time in participants treated with emicizumab who had FVIII inhibitors (*n* = 206) in HAVEN 1, 2, and 4 (Figure 5). As the assay has an upper limit of 45 CBU/mL, it is possible that the actual mean titer is higher. The proportion of participants with a FVIII inhibitor titer of <0.6 CBU/mL at week 25 was 8.5%, increasing to 14.5% at week 49, 17.2% at week 73, 17.9% at week 121, and 30.8% at week 181. No *de novo* inhibitors developed in participants without FVIII inhibitors in HAVEN 3 and 4.

### 3.7 Impact of FVIII inhibitors and age on PK

**Figure 6:**
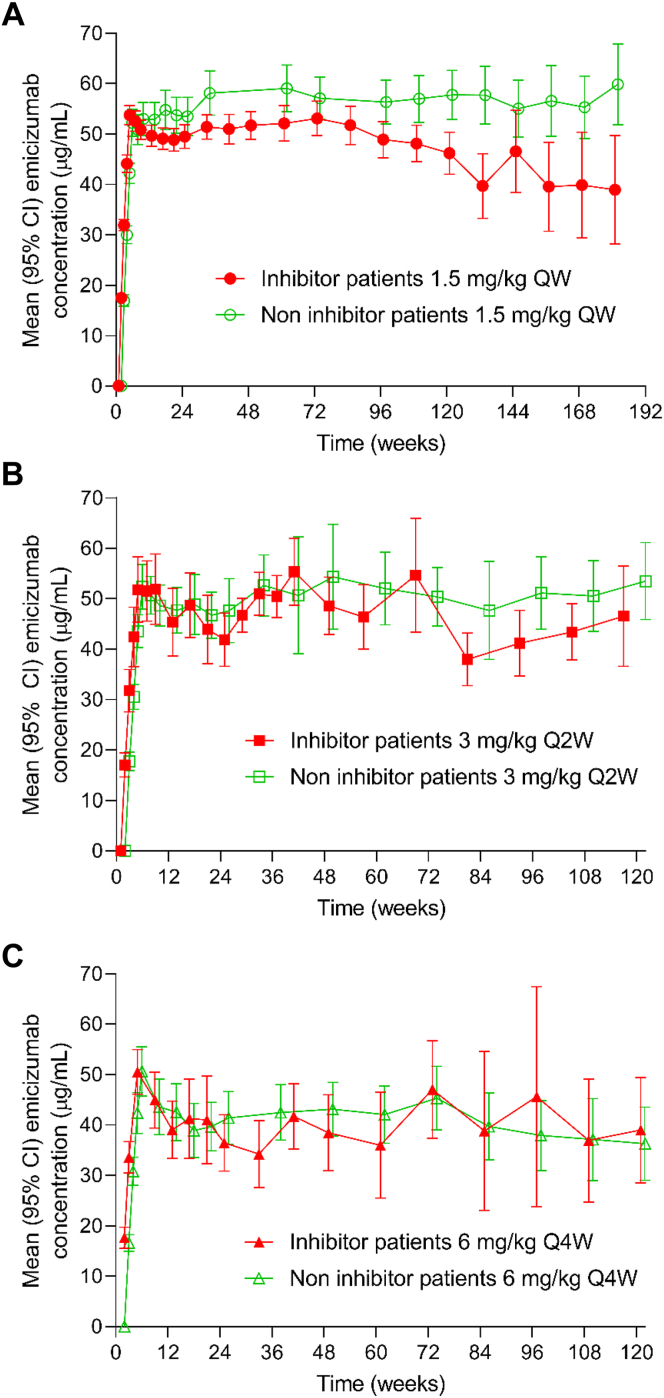
Emicizumab concentration over time by factor VIII inhibitor status for participants receiving (A) emicizumab 1.5 mg/kg once weekly (QW), (B) 3 mg/kg every 2 weeks (Q2W), and (C) 6 mg/kg every 4 weeks (Q4W). This analysis includes data from different studies, so not all participants have been tested at each time point.

**Figure 7:**
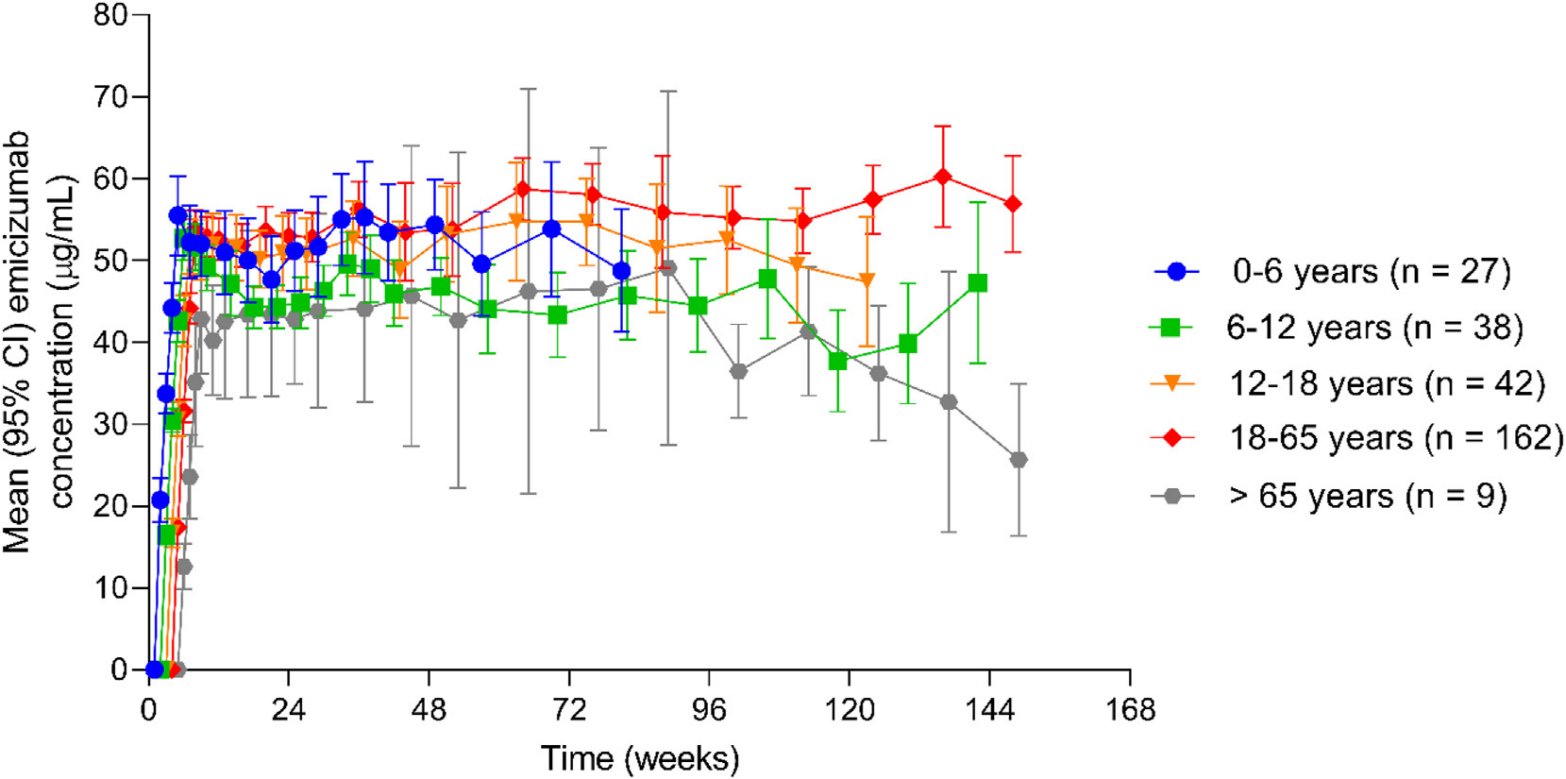
Emicizumab concentration over time according to age category for participants who received the once weekly dosing regimen. Mean data were presented when participants with available data represented ≥30% of the total participants in a given age group. CI, confidence interval.

Exploratory subgroup analysis of emicizumab PK was performed in people with HA with and without FVIII inhibitors and in different age groups. PK profiles of emicizumab were similar in those with or without FVIII inhibitors who received emicizumab 1.5 mg/kg weekly, 3 mg/kg every 2 weeks, or 6 mg/kg every 4 weeks (Figure 6). PK profiles of emicizumab were similar in the groups aged 0 to 6 years (*n* = 27), 6 to 12 years (*n* = 38), 12 to 18 years (*n* = 42), 18 to 65 years (*n* = 162), and >65 years (*n* = 9) who received weekly maintenance dosing (Figure 7). There was no significant difference in the PK profiles of emicizumab irrespective of FVIII inhibitor status or age.

## 4 Discussion

In samples collected throughout the HAVEN 1 to 4 clinical trials, emicizumab demonstrated sustained PK and PD and improved coagulation parameters without affecting safety biomarkers.

Emicizumab dosing regimens were initially selected based on population PK/efficacy modeling to rapidly achieve and maintain therapeutic emicizumab trough concentrations [5]. In this study, in line with the predictions of the PK model, mean emicizumab trough concentrations slightly greater than 50 μg/mL were achieved at the end of the loading dose period in HAVEN 1 to 4 and were maintained for the follow-up duration (up to 3.5 years). These concentrations provided sustained therapeutic exposure to emicizumab for the majority of participants, as demonstrated by 56% to 90% of participants who did not report any treated bleeds in the HAVEN 1 to 4 studies [[8], [9], [10], [11],^15^]. In HAVEN 1, the previously observed emicizumab exposure at >50 μg/mL [16] was sustained for an additional 26 months with the maintenance dose. As expected, based on the mechanism of action of emicizumab and its lack of shared sequence homology with FVIII, PK profiles of emicizumab were similar regardless of FVIII inhibitor status, and there was no development of *de novo* FVIII inhibitors over the entire follow-up period. Low immunogenicity of emicizumab has been previously described [24]. In the previous analysis, a total of 16 participants in HAVEN 1 to 4 developed antidrug antibodies (ADAs) to emicizumab, and in 10 of them, ADAs were neutralizing *in vitro*. The presence of neutralizing ADAs was associated with faster drug elimination in 3 participants, one of whom discontinued treatment for lack of efficacy [24]. As predicted by the PK models [5,25], age over the range enrolled in these studies (oldest participant enrolled was aged 77 years) did not affect PK overall, although exposure tends to decrease in the elderly [18].

Effects of emicizumab on the coagulation cascade were monitored in the HAVEN 1 to 4 studies via various PD markers. Similar to previous observations [3,16], aPTT was shortened to below the upper limit of the reference range after the first dose of emicizumab when concentrations had not yet reached therapeutic levels. Unlike FVIII, emicizumab does not require a rate-limiting activation step but instead directly mimics the cofactor activity of activated FVIII in FX activation by FIXa. Hence, the observed shortening effect of emicizumab on aPTT, greater than that of FVIII agents, was expected [26].

Conventional one-stage FVIII activity assays, which rely on aPTT-based clotting time analysis, report artificially elevated FVIII activity in the presence of emicizumab [26]. Thus, a chromogenic FVIII activity assay with human factors, which is sensitive to emicizumab, was used to assess the PD effect of emicizumab. Activity obtained using the human chromogenic FVIII assay provides surrogate FVIII-like activities, which do not represent a true FVIII equivalence [19,27], but can be used to measure the relative PD effect of emicizumab. Mean FVIII-like activity increased during the loading dose period to approximately 25 IU/dL and was sustained thereafter with the weekly, every 2 weeks, and every 4 weeks maintenance doses until week 109.

FVIII-like activity observed with the weekly maintenance dosing was similar to what was previously reported in the smaller HAVEN 1 population with a shorter follow-up [16]. An increase in FVIII-like activity was observed after week 120 with all dosing regimens, and a decrease observed after week 168; this is considered an assay-related artifact as the increase and later decrease coincided with changes in the human chromogenic FVIII assay kit lots, and there were no concomitant changes in emicizumab concentration or TG peak height. The chromogenic FVIII assay was calibrated with a FVIII-containing plasma calibrator, and there was no difference between the assay kit lots when samples containing FVIII were measured, again highlighting that FVIII activity reported for people with HA treated with emicizumab with this assay cannot be compared with, or interpreted as equivalent to, FVIII activity reported in those treated with FVIII. The levels of FVIII within the sample to be measured are normally the limiting factor in this assay. In contrast, due to the different biochemical properties of emicizumab and FVIII [7,19], measured emicizumab activity is dependent on the amount of FIXa available as emicizumab concentrations exceed those of FIXa in the assay. It has been previously reported that levels of FIXa vary between chromogenic assays from different manufacturers and may differ in a lot-to-lot manner in chromogenic assay kits that use the same reagents; some assays are intended for research use only and consequently generate different FVIII-like activity values for emicizumab-containing samples [28]. Our data confirm that lot-to-lot variability is an important consideration if human chromogenic FVIII assay is used to measure samples containing emicizumab and that measurements performed with different kit lots are not necessarily comparable, especially when the calibrator (FVIII) and the analyte (emicizumab) are not identical. To enable reliable measurement of emicizumab, a dedicated calibrator and controls for emicizumab (*r*^2^ Diagnostics) have been developed and can be used with a modified 1-stage assay [29]. The emicizumab calibrator has been tested across different modified 1-stage assay reagents and in combination with a human chromogenic FVIII assay [30]. This assay has shown good technical performance, including lot-to-lot reproducibility, and can be used to measure emicizumab levels in clinical situations where checking drug concentrations is desired [31]. Alternatively, the human chromogenic assay can be calibrated with the emicizumab calibrator, as previously demonstrated [30].

Importantly, some studies have elucidated the approximate FVIII level in people with severe HA and inhibitors receiving emicizumab to determine its FVIII equivalency. A study by Kizilocak et al. [32] assessed FVIII levels and TG in people with mild or moderate HA using a linear regression to model FVIII levels as a function of the TG assay parameters and to make a calibration curve of FVIII levels vs peak thrombin and endogenous thrombin potential. People with severe HA with FVIII inhibitors on emicizumab had TG performed in the same manner and their peak thrombin and endogenous thrombin potential results were placed on the calibration curve to calculate their FVIII equivalency of emicizumab by TG. The results of this study showed all participants on emicizumab had a FVIII equivalency of emicizumab by TG of >10%, with most having levels of 10% to 40% (some even higher), suggesting that people with severe HA receiving emicizumab have similar TG to people with mild HA [32].

In this pooled analysis of HAVEN 1 to 4 participants, mean TG peak height increased to approximately 116 nM during the loading doses and was maintained above 116 nM throughout the follow-up period regardless of dosing regimen. Interindividual variability in TG was higher than that in emicizumab concentration or FVIII-like activity. This could be expected as large variability in TG has also been observed when analyzing healthy individuals [33]. The observed TG data are consistent with previous HAVEN 1 data [16], where TG peak height was sustained at approximately 110 nM, which corresponds to 20% to 30% of the TG peak height in healthy people (316-488 nM) using similar assay conditions [4]. Analyses of hemostatic potential using global assays in small, real-world cohorts of people with severe HA treated with emicizumab have also demonstrated improvements in TG to levels similar to those observed in people with a mild HA phenotype [32,34]. Although TG assays are good tools to confirm improvements in the hemostatic profile of people with HA in response to treatment, it is currently not clear how well the TG potential *in vitro* correlates with clinical outcome [35,36]. In addition, similar to the human chromogenic FVIII activity assays, emicizumab activity depends on the amount of FIXa present in the TG assays, which thus cannot be used to determine the true FVIII equivalence of emicizumab [28]. In animal models, emicizumab at therapeutic levels (26-61 μg/mL in primates) was estimated to be equivalent to 10% to 20% FVIII in a primate model of acquired HA [37] and in a semihumanized mouse model for FVIII deficiency [38]. Although it is not possible to derive FVIII equivalence of emicizumab from any single laboratory test or model, the available evidence overall supports that emicizumab converts a severe bleeding phenotype to a mild phenotype.

As expected, based on the emicizumab structure and mechanism of action, there was generally no difference in PK or PD or the PK/PD relationships between people with HA with and without FVIII inhibitors. In those without FVIII inhibitors, the add-on effect of treatment with FVIII products was clearly visible in FVIII-like activity measurements and TG, where high values were observed in some individuals even at baseline prior to the first dose of emicizumab. PK/PD relationships were similar to those previously observed in HAVEN 1, except for the add-on effect of FVIII treatment, which was not seen in HAVEN 1, where all participants had FVIII inhibitors. It is important to note that cases of thrombotic microangiopathy and thrombotic events have been reported when >100 U/kg/24 h of aPCC were administered for ≥24 hours concomitantly with emicizumab [8]. Owing to this synergistic effect, the concomitant use of emicizumab with aPCC should only be initiated if no other options are available.

As emicizumab does not drive activity in the tenase reaction with bovine proteins FIX and FX, the CBA allows measurement of FVIII inhibitors despite the presence of emicizumab. In people with HA with FVIII inhibitors, the proportion of participants with a titer of <0.6 CBU/mL increased with time—from 8.0% at baseline to 14.5% at week 49, 19.4% at week 109, and 30.8% at week 181—likely due to a lack of exposure to FVIII. Overall, 31 of 206 (15.0%) participants with inhibitors had a titer of <0.6 CBU/mL at their last observation; no *de novo* FVIII inhibitors were observed in people with HA without inhibitors during emicizumab treatment.

Based on the relatively low binding affinities to FIX and FX *in vitro* [7], emicizumab was not predicted to affect their plasma concentrations, and no change in FIX and FX antigen levels was observed during emicizumab treatment. Emicizumab was also not anticipated to affect the extrinsic pathway of the coagulation cascade, which was confirmed by the lack of significant effect observed on PT. As expected, based on the favorable safety profile of emicizumab in the HAVEN 1 to 4 trials [[8], [9], [10], [11],^15^], no changes were observed in the steady-state levels of exploratory safety markers of coagulation activation (D-dimer, prothrombin fragment 1+2, and fibrinogen) during emicizumab treatment. This is consistent with previous reports and the inability of emicizumab to activate coagulation in the absence of an initiating signal [16].

There were some limitations to these analyses. Longitudinal analysis can be complicated by variability in assay reagents, as the same kit lots are not available for such long periods and assay shelf-lives can be shorter than the study duration. As discussed above, there is no laboratory assay that allows direct comparison of the efficiency or equivalence of emicizumab with FVIII products [28]. Sample size is a limiting factor due to the relatively small number of participants in some of the subgroup analyses performed.

In conclusion, emicizumab dosing weekly, every 2 weeks, or every 4 weeks in HAVEN 1 to 4 demonstrated sustained PK and PD for >3 years and comparable PK/PD characteristics as observed in people with HA with FVIII inhibitors who received the weekly dosing regimen in HAVEN 1. FVIII inhibitor status or the age of the population did not, in general, have an impact on emicizumab PK or PD. Emicizumab did not significantly affect FIX or FX plasma antigen levels, PT, or concentrations of exploratory safety markers of coagulation activation (D-dimer, prothrombin fragment 1+2, and fibrinogen). Hence, emicizumab improved coagulation parameters without showing abnormal signs of interaction with FIX and FX or affecting safety biomarkers.
